# Health risk assessment of heavy metal pollution in a soil-rice system: a case study in the Jin-Qu Basin of China

**DOI:** 10.1038/s41598-020-68295-6

**Published:** 2020-07-13

**Authors:** Bin Guo, Chunlai Hong, Wenbin Tong, Mingxing Xu, Chunlei Huang, Hanqin Yin, Yicheng Lin, Qinglin Fu

**Affiliations:** 1 Institute of Environment, Resource, Soil and Fertilizer, Zhejiang Academy of Agricultural Sciences, Hangzhou, 310021 China; 2 Technological Innovation Center for Arable Land Assessment and Restoration of Ministry of Natural Resources, Hangzhou, 311203 China; 3 Qujiang District Agricultural and Rural Burea, Quzhou, 324022 China

**Keywords:** Environmental impact, Environmental impact

## Abstract

A regional field survey of a total of 109 pairs of soil and rice samples was conducted to evaluate the health risks posed by heavy metals in the Jin-Qu Basin, China. The studied soils are characterized by acid (pH in mean level of 5.5), carbon rich (soil organic matter in mean of 33.6 g kg^−1^) and mainly contaminated by Cd (42.2% samples exceeded the standard value of 0.3 mg kg^−1^ (GB15618-2018)). The spatial distributions of Cd, Pb and Zn exhibited similar geographic trends. 34% and 30% of the rice samples containing Cd and Pb exceeded the threshold value of 0.2 mg kg^−1^ (GB2762-2017), respectively. The risk estimation of dietary intake had a target hazard quotient value of Cd of 0.918 and a hazard index value for rice consumption of 2.141. Totally, Cd and Pb were found to be the main components contributing to the potential health risks posed by non-carcinogenic effects for local inhabitants.

## Introduction

Over the past decades, heavy metals contamination in agricultural soil has drawn international attention^1^. Apart from being naturally introduced through parent materials, the elevation of heavy metals in farmland soil mainly results from anthropogenic activities such as wastewater irrigation, unqualified fertilizer input, deposition of airborne metals through smelting and fossil fuel combustion^2^. Intensifying processes of soil contamination has been posed potential risks to the environment and to safe crop production^3^.

Rice is a major staple food for the Chinese population. The average level of rice consumption in China is 238 g^−1^capita^−1^ day^−1^^4^, which is 61% higher than the global average level of 148 g^−1^capita^−1^ day^−1^^5^. Due to its rapid growth and high biomass, rice is efficient in assimilation of heavy metals from soils^6^. The chronic intake of heavy metals through rice consumption can cause serious effects on psychotic disorders and irretrievable damage to human health. For example, the hazard values of Cd and Pb in rice are both 0.2 mg kg^−1^ in China (GB 2762-2017). Long term consumption of the contaminated rice has been widely recognized in causing Itai-Itai symptom for Cd^7^ and intelligence quotient for Pb^8^. Hence, the transference of heavy metals into a soil–rice system has been raised as an issue of concern in China^3^. Numerous surveys of specific areas (e.g., mining areas) and pot experiments have been performed regarding how mine drainage and soil conditions affect the accumulation of heavy metals in rice grains^9^. However, such studies may not predict the spatial variability and spatial relationships of heavy metals at regional scales. Therefore, surveys of heavy metals found in soil-rice systems involving regional field investigations should be encouraged due to their greater significance to agronomic practices.

Zhejiang is one of the richest and most developed provinces in China, providing roughly 6.24% of the country's GDP and housing to 56.6 million inhabitants^10^. A national soil pollution survey conducted in 2014 shows that roughly 15% of the agricultural land in China has been polluted with heavy metals^11^. Due to inadequate land resources in this province, most contaminated agricultural land is still being used for crop production. This has spurred considerable concern due to corresponding potential risks to human health and the environment. The statistical and geostatistical characterization of heavy metals has been surveyed in this province, such as Hangzhou^12^, Fuyang^13^, and Wenling^14^. The surveys were better accuracy with traditional field sampling and chemical analysis followed by the ordinary Kriging mapping method. Recently, remote sensing-based hyperspectral technology with time- and cost-efficient merit has been recommended to identify the spatial distribution of heavy metals^15,16^. However, many studies still preferred to the traditional method due to its more accuracy and reliability for the survey of heavy metal contamination.

The previous studies in Zhejiang province were mainly focused on urban–rural transitional areas that affected by irresponsible anthropic inputs and disposal of electric wastes. Due to the multi-anthropogenic sources, it is difficult to disclose the origins of trace elements and to characterize their spatial patterns^12^. Accordingly, there were large variations of the heavy metal contents of rice samples collected even within the same producing areas. For example, studies in 2008^14^ and 2010^17^ showed that 31% and 9.4% of the rice grains taken from 13 and 96 paddy soils in Wenling, respectively, were contaminated by Cd. However, the survey of the major rice producing catchments in Zhejiang province, including the high-risk paddy land in the Jin-Qu Basin, is still lacking. This area is mainly influenced by nonferrous metal smelting operations and irrigation with contaminated surface water, which is comparable of the surveys of Hunan, a province that hosts many mining factories^3^.

Therefore, the main objectives of this study were to (1) investigate heavy metals pollution (Cd, Pb, Ni, Cr, Zn, and Cu) according paddy soils and rice grains in the Jin-Qu Basin and (2) the effects of soil parameters (e.g., pH and organic matter) on heavy metals transfer from soils to rice grains and (3) to evaluate the health risks posed by heavy metals as a result of rice consumption. Our findings may guide policies that can protect paddy soils in this region and that can serve as a basis for comparisons to other areas both in China and worldwide.

## Results and discussion

### Heavy metals accumulation in soils

Table 1 presented the summary statistics for heavy metal concentrations together with soil organic matter (SOM) contents and pH values of paddy soils from Jin-Qu Basin in Zhejiang province. In addition, average background values for Zhejiang soils^18^ and Chinese soil quality standards (GB15618-2018)^19^ were compared in Table 1. Soil pH and SOM levels ranged from 4.48 to 7.38 and from 1.59 to 3.36%, respectively, with average values of 5.52 and 3.36%, respectively, characterizing acid and carbon rich properties of paddy soils in this region. Large organic carbon stocks observed in paddy soils were attributable to anaerobic conditions and high inputs of organic matter. Soil acidification was a major problem observed in southern paddy soils in China. Apart from natural weathering soil minerals, the overuse of N fertilizers contributes substantially to soil acidification in China^20^.

**Table 1:** Descriptive data of heavy metals in paddy soils from Jin-Qu basin of Zhejiang province (n = 109).

	Cd (mg kg^−1^)	Pb (mg kg^−1^)	Ni (mg kg^−1^)	Cr (mg kg^−1^)	Zn (mg kg^−1^)	Cu (mg kg^−1^)	SOM (%)	pH
**Data**								
Max	1.68	108.5	100.4	175.3	251.5	77.3	6.15	7.38
Min	0.06	5.0	0.3	2.8	11.9	1.0	1.59	4.48
Median	0.32	29.3	11.6	19.8	93.9	14.3	3.23	5.17
**Statistics**								
Mean	0.37	32.1	15.2	24.2	98.7	16.7	3.36	5.22
SD	0.249	11.9	15.2	22.6	54.4	11.1	0.96	0.64
CV	68%	37%	100%	93%	55%	67%	29%	12%
K-S (p)	0.009	0.010	0.000	0.001	0.353	0.045	0.623	0.001
**References**								
Background^a^	0.17	25.6	22.3	54.6	69.0	17.8	–	–
Guidelines^b^	0.30	80	60	250	200	50	–	–
Exceeding	42.2%	0.9%	2.8%	0	3.7%	1.8%	–	–
**Mean values of different counties**								
Dongyang (n = 38)	0.18	24.9	11.0	19.1	68.8	9.5	3.66	5.25
Jiangshan (n = 33)	0.44	31.5	28.9	43.9	127.5	29.0	4.14	5.38
Longyou (n = 38)	0.55	42.1	17.3	24.2	113.1	21.9	2.61	5.23

^a^Zhejiang Soil Survey Office. Zhejiang Soils (1994).

^b^Soil environmental quality of risk control standard for soil contamination of agricultural land (GB15618-2018).

The mean level of Cr (24.2 mg kg^−1^) was measured as only 43% of the background value recorded for Zhejiang province^18^. The average top soil Pb, Ni, and Cu levels were comparable to the corresponding background values, indicating that these metals were derived from an inherent pattern of spatial heterogeneity. In contrast, mean concentrations of Cd (0.37 mg kg^−1^) and Zn (98.7 mg kg^−1^) were recorded as 117% and 43% higher than background values for Zhejiang province. Moreover, Cd concentrations measured in 49 soil samples were found to exceed the standard value (0.3 mg kg^−1^), accounting for 42.2% of the soil samples followed by Zn (200 mg kg^−1^) being found in 4 soil samples (3.7%), Ni (60 mg kg^−1^) being found in 3 soil samples (2.8%), Cu (50 mg kg^−1^) being found in 2 soil samples (1.8%), and Pb (80 mg kg^−1^) being found in 1 soil sample (0.9%). These results indicated that different point-pollution sources might exist in the Jin-Qu Basin.

The coefficient of variation (CV) is a standardized measure of the dispersion of a probability or frequency distribution. It represents the ratio of the standard deviation to the mean. In this study, all metals of the CV were found to exceed at value of 50% except in the case of Pb (37%) (Table 1). Furthermore, the normality of heavy metal concentrations was determined through a Kolmogorov–Smirnov (K-S) test by generating cumulative probability plots for two distributions and finding the distance along the y-axis for a given x values between the two curves. All of the studied metals were strongly skewed and did not pass the normality test (p < 0.05) except for Zn (Supplementary Fig. S1), revealing the non-normal distribution of raw data for the heavy metals. Non-normal concentrations of heavy metals such as Cd together with the extensive variable distribution observed in the Jin-Qu Basin suggested that heavy metals in the paddy fields might be increasing as a result of various anthropogenic activities occurring due to rapid economic development^21^.

The Pearson's correlation analysis was used to identify the relationships of the trace elements and soil properties (Table 2). Negative correlations between soil pH and Cd, Ni and Zn were found, which has been well documented in previous studies^22,23^. Soil pH was considered to be the most influential factors that shape heavy metals transfer. Low soil pH led to metal solubility by altering the chemical speciation^26^. SOM was another important soil property with dual effects on heavy metals mobility^24^. It infiltrated organic chemicals into soil solutions, which might serve as chelates and increase metals availability in plants. However, it might also reduce the bioavailability of heavy metals through adsorption or by forming stable complexes with humic substances^25^. The complex effects of SOM led to uncertain results on heavy metals solubility relative to those on soil pH.

**Table 2:** The Pearson correlation between the concentrations of heavy metals, soil pH and soil organic matter.

	Cd	Pb	Ni	Cr	Zn	Cu	SOM	pH
Cd	1							
Pb	0.557**	1						
Ni	0.504**	0.428**	1					
Cr	0.242*	0.412**	0.838**	1				
Zn	0.389**	0.504**	0.294**	0.259*	1			
Cu	0.507**	0.759**	0.527**	0.431**	0.623**	1		
SOM	− 0.142	− 0.105	0.057	0.125	0.007	− 0.093	1	
pH	0.222*	− 0.121	0.228*	0.057	− 0.222*	− 0.071	− 0.036	1

*p < 0.05, **p < 0.01; significant correlations (two-tailed).

The positive relationships between Cd, Pb, Ni, Cr, Zn and Cu were all highly significant (p < 0.01 or p < 0.05). The close correlations between Cu and Pb (R^2^ = 0.759) and between Ni and Cr (R^2^ = 0.838) coincide with data collected from plains in Hangzhou (Cu and Pb, R^2^ = 0.80)^12^ and Huanghuai in China (Ni and Cr, R^2^ = 0.95)^26^, respectively. The analysis indicated that, to some extent, these trace elements might be originated from a common pollution source.

PCA was made to further identify the possible source of the heavy metals and their relationships with soil properties. PCA is a powerful pattern recognition tool that can explain the variance of a large dataset of inter-correlated variables with a smaller set of independent variables^27^. Three principal components (PC1, PC2, and PC3) were extracted with eigenvalues > 1 when applying varimax rotation, accounting for the majority (75.3%) of the total variances (Table 3). Consequently, the correlation between the loadings of the three principal components and heavy metals, pH, SOM was analyzed in this study. According to the results, Cd, Pb, Ni, Cr, Zn and Cu was positive related to the PC1 (0.663–0.860), and the same as noted in pH for PC2 (0.757) and SOM for PC2 (0.788). PC1, the most important component, explained 39.8% of the total variances and was characterized by high coefficient factors of the tested metals. This reflected that Pb, Ni, Cr, and Cu in the soils might be originated from the weathering of the parent material and subsequent pedogenesis, because the levels of these metals are similar to the background values. In contrast, Cd and Zn might mainly result from anthropogenic input, since the mean concentrations of these two elements were 2.18 and 1.43 times higher than the background values (Table 1). PC2 explained 17.8% of the total variances, mainly including pH and reflecting the influence by soil acidity. PC3 explained 17.7% of the total variance and was dominated by high loadings of SOM, reflecting their association with soil organic matter.

**Table 3:** Principal component factor scores for heavy metals of soil from Jin-Qu basin of Zhejiang province.

Component	Rotation sums of squared loadings		
	Total	Variance (%)	Cumulative (%)
1	3.19	39.8	39.8
2	1.42	17.8	57.6
3	1.41	17.7	75.3

Element	Rotated component matrix		
	PC1	PC2	PC3
Cd	0.705	0.072	− 0.412
Pb	0.817	− 0.277	− 0.079
Ni	0.790	0.490	0.132
Cr	0.695	0.418	0.369
Zn	0.663	− 0.455	0.119
Cu	0.860	− 0.243	− 0.035
SOM	− 0.053	0.234	0.788
pH	0.029	0.757	− 0.462

### Spatial distribution of heavy metals in paddy soils in the Jin-Qu Basin

**Figure 1:**
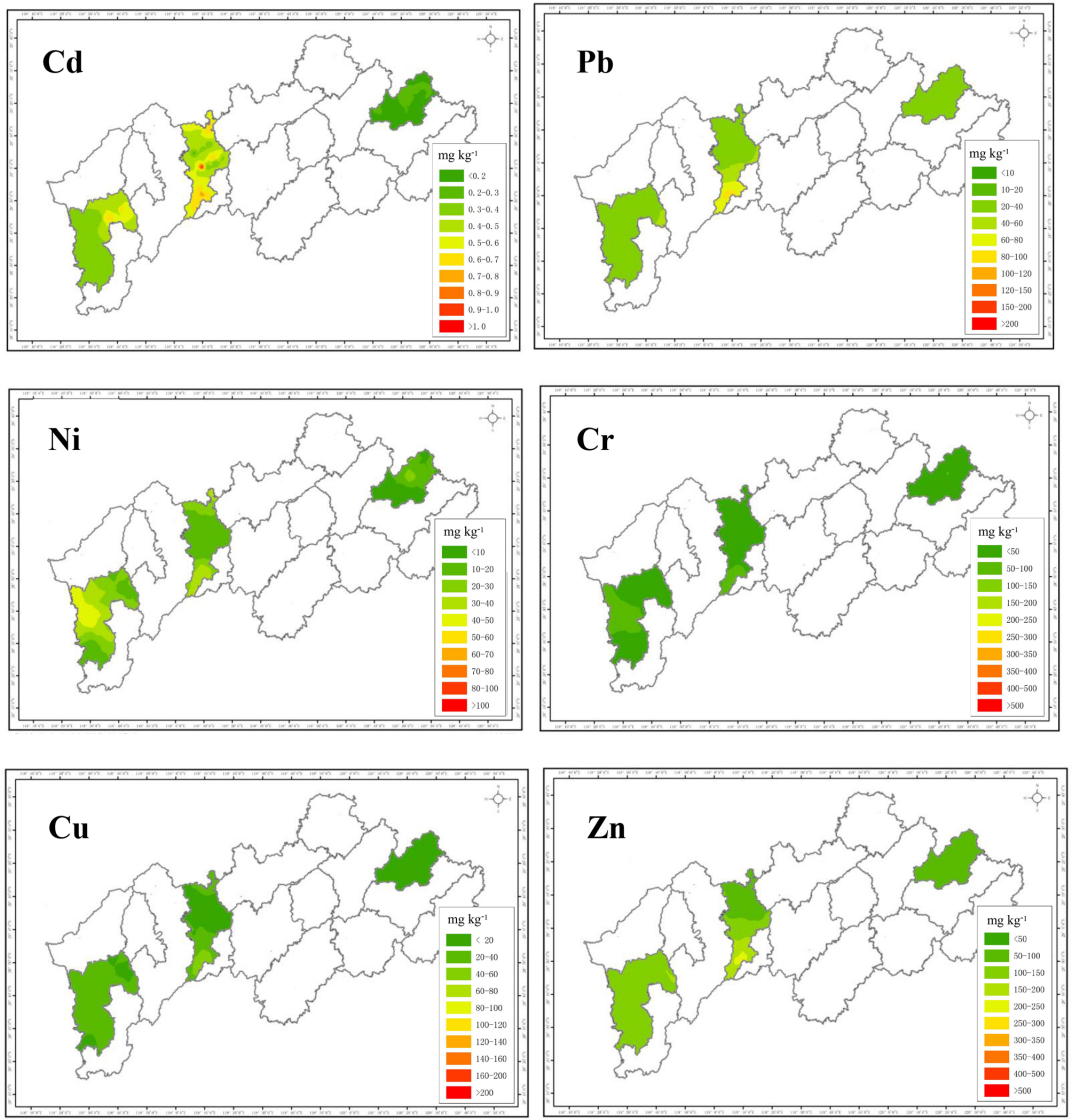
Spatial distribution of heavy metal contents in surface soils in Jin-Qu basin (The map was created by ARCGIS 10.5, https://developers.arcgis.com/).

Figure 1 showed the spatial distribution of heavy metals in paddy soils in the Jin-Qu Basin. Samples were drawn from three typical agricultural areas: the eastern (Dongyang county), central (Qujiang county), and western (Jiangshan county) sections. Spatial distribution maps for Cd, Pb and Zn showed similar geographic trends with the lowest concentrations observed in the east, with moderate concentrations observed in the west, and with the highest concentrations observed in the central region. The common spatial distribution of the three metals indicated that they had the same pollution sources, which was consistent with the PCA analysis (Table 3). The topography of the central area was characterized by hilly mountainous terrain in the periphery and by plains in the centre. The mining industry in this area, such as Quzhou uranium mine^28^, Quzhou Shangfang mine (Pb/Zn)^29^, Jinhua Suichang mine (gold)^30^, etc., had expanded rapidly to meet the growing demands for mineral raw materials during fast industrial transition. Inconveniently, most of nonferrous metal deposits were located within the major rice production regions, which directly or indirectly resulted in leaching of large quantities of effluent along with heavy metals into nearby rivers and streams. This has brought serious damages to the environment and caused heavy metals accumulate in the paddy soils and crops. Due to the various of mining sources and transporting modes, the formation and dispersion processes of heavy metal contamination were still hardly differentiated. The spatial distribution mapping of heavy metal contamination could be improved using GIS enhanced multiple linear regression in the future investigation^31^.

Chemical speciation heavily influenced the mobility of metals in soil. It had been suggested that the bioavailable fraction of metals rather than total heavy metals levels was needed to assess the level of phytotoxic risk^32^. However, a wide variety of bioavailable extraction procedures for soil analysis involving different sequences of extractants such as MgCl_2_, CaCl_2_, NH_4_Cl, EDTA, CH_3_COOH, etc. had been developed^33^. In this study, the first extraction step of the BCR method (0.11 M CH_3_COOH) was used, as this method has been widely adopted as a standardize sequential extraction schemes for the determination of bioavailable elements in soils^34^. The means of CH_3_COOH (HAc)-extractable metal concentrations in soil samples were recorded as Zn > Pb > Cu > Ni > Cr > Cd (Table 4), echoing the ordering of total soil concentrations. Significant relationships were found between HAc-extractable forms and total levels such as Cd (0.7676, p < 0.01) and Pb (0.3253, p < 0.01). These results suggested that the mobility of heavy metals was determined by total content levels.

**Table 4:** Descriptive data of HAC-extractable metals (mg kg^−1^) in paddy soils from Jin-Qu basin of Zhejiang province (n = 109).

	Cd (mg kg^−1^)	Pb (mg kg^−1^)	Ni (mg kg^−1^)	Cr (mg kg^−1^)	Zn (mg kg^−1^)	Cu (mg kg^−1^)
**Data**						
Max	0.525	2.80	3.41	1.34	9.32	3.27
Min	0.027	0.31	0.10	0.10	1.24	0.04
Median	0.089	1.11	0.42	0.37	3.68	0.46
**Statistics**						
Mean	0.072	0.91	0.34	0.34	3.26	0.35
SD	0.074	0.58	0.39	0.17	1.89	0.46
CV	83.1%	52.2%	93.0%	45.9%	51.3%	98.5%
**Regression analysis of HAc-extractable to total content**						
R^2^	0.7676	0.3253	0.3608	0.3722	0.2404	0.5561
Significant level	p < 0.01	p < 0.01	p < 0.01	p < 0.01	p < 0.01	p < 0.01

### Heavy metals accumulation in rice grains

As noted in Table 5, all of mean concentrations of the heavy metals belowed the levels outlined by Chinese national standards on pollutants in food for food safety (GB 2762-2017). However, 34%, 30% and 6% of the rice samples contained Cd, Pb, and Cr levels in excess of standard levels of 0.2 mg kg^−1^, 0.2 mg kg^−1^and 1.0 mg kg^−1^, respectively. The highest concentrations of Cd, Pb, and Cr were recorded as 1.094, 0.483, and 1.34 mg kg^−1^, respectively.

**Table 5:** Comparison of the concentrations of six heavy metals in rice grain (mg kg^−1^) in this research with the data from some previous studies.

	Cd (mg kg^−1^)	Pb (mg kg^−1^)	Ni (mg kg^−1^)	Cr (mg kg^−1^)	Zn (mg kg^−1^)	Cu (mg kg^−1^)
**Data (n = 86)**						
Max	1.094	0.483	14.55	1.34	58.67	6.75
Min	0.028	0.048	0.28	0.05	19.22	2.33
Median	0.233	0.175	1.14	0.38	29.64	4.17
**Statistics**						
Mean	0.163	0.148	0.88	0.31	27.67	4.04
SD	0.206	0.094	1.57	0.25	7.89	0.91
CV	88.1%	53.5%	137.8%	66.9%	26.6%	21.8%
**References**						
Guidelines ^a^	0.2	0.2	–^b^	1.0	–	–
Exceeding	34%	30%	–	6%	–	–
**Mean values and range of other areas**						
Zhejiang Province^c^ (n = 248)	0.037	0.060	–	–	–	–
	LOD-0.112	0.005–0.220	–	–	–	–
Wenling (n = 219)	0.132	–	0.223	–	17.4	2.46
	0.011–2.50	–	0.014–0.959	–	10.3–31.4	0.580–5.05
Changshu (n = 155)	0.019	0.171	–	0.292	19.1	3.84
	LOD-0.201	LOD-0.957	–	0.024–0.742	9.10–28.3	0.87–7.77

^a^Chinese national standards for food safety limit of pollutants in food (GB 2762-2017).

^b^The threshold value is not defined.

^c^Huang et al.^43^.

^d^Li et al.^21^.

^e^Hang et al.^35^.

Cd is one of the most toxic and mobile soil elements. Health risks of consuming Cd-contaminated rice were of particular concern since rice consumption accounts for the majority of calories consumed by southern Chinese populations^3^. Exposure to Cd has adverse health effects on the pulmonary, cardiovascular, and musculoskeletal systems as well. In the present study, Cd concentrations in rice grains were found to range from 0.028 to 1.094 mg kg^−1^ with a mean of 0.163 mg kg^−1^. Mean Cd concentrations belowed the standard level of 0.2 mg kg^−1^ but still were much higher than those in commercial rice collected from Zhejiang province (0.037 mg kg^−1^)^23^ and Changshu (0.019 mg kg^−1^)^35^, a major rice producing area in the Yangtze River Delta, China. This shows that levels of Cd contamination in rice grains in the Jin-Qu Basin are higher than those observed in other cultivation areas, which may be influenced by nonferrous metal plants present in this area. Similarly, field survey results on nonferrous metal production zones in Hunan province, China show that levels in 65% of the studied rice samples exceed national food standards limits^36^.

Pb is classified as an element carcinogenic to humans. Chronic exposure of Pb causes headaches, convulsions, paralysis, and neurological damage especially in young children. Pb concentrations in the rice samples ranged considerably from 0.048 to 0.483 mg kg^−1^ with a mean value of 0.148 mg kg^−1^, echoing results found for the other regions impacted by mining (0.185 mg kg^−1^, n = 122)^8^. These levels were quite high compared to those of rice samples collected from markets in China (0.037 mg kg^−1^, n = 88)^8^. In fact, 30% of the rice samples were found to exceed the safe threshold level of 0.2 mg kg^−1^. The long-term consumption of Pb-contaminated rice presented high risks to human health in the Jin-Qu Basin. Pb might be introduced through atmospheric deposition during mining and smelting production, as no Pb soil contamination was observed in the region (Table 1). This finding complemented a study conducted in a base-metal mining area of Hunan province, China that dust and particulate material deposition constituted the main source of Pb introduction into rice grains^37^.

The accumulation of heavy metals in the paddy soils poses a threat to human health through food chain. The Bio-accumulation factor (BAF) is an index for evaluating the transfer potential of a metal from soil to rice grains. The value of Cd for rice grains (0.250) was much higher than other test metals (Supplementary Fig. S2), which is consistent with the previous reports^38,39^. This finding indicated the occurrence of relatively high levels of some metals in paddy soils, such as Cr and Pb, did not necessarily result in excessive accumulation of the metals in rice grains. In fact, the uptake by rice root and subsequent accumulation in grains was affected by many soil factors, including acidity, organic matter, clay minerals, and Fe/Mn oxides^3^. Compared with other metals, Cd could be more easily released from the solid phase into the pore water. Hence, the accumulation of Cd in rice was often controlled to greater extent by its total content in the soil than the other metals^3^. As shown in the present study, Cd presented the highest levels of bioavailability of the tested metals. Significantly positive correlations with Cd concentrations were found for the soil and rice samples (R^2^ = 0.7676, p < 0.01), which were much higher than the other five metals.

### Heavy metal risks to human health via rice consumption

Although there are many pathways of human exposure to heavy metals, such as drinking, soil ingestion, dermal contact and inhalation routes^43^, the dietary consumption has been identified as the major pathway which contributes over 90% of the health risk^40,41^. Therefore, this study was particularly focused on the heavy metal risks via rice consumption in this region. Daily intake (EDI) levels of Cd, Pb, Ni, Cr, Zn and Cu metals through rice consumption were estimated at 0.92, 1.38, 4.5, 1.5, 116.5, and 16.4 μg kg^−1^ day^−1^, respectively (Table 6). Accordingly, potential health risks posed by Cd, Pb, Ni, Cr, Zn and Cu as indicated by THQ values were recorded as 0.918, 0.394, 0.112, 0.001, 0.388, and 0.328, respectively. The THQ of Cd was close to a value of 1, suggesting that rice consumption might pose serious health risks to local populations. This result was similar to the report that the Cd and Pb THQ values were 1.03 and 0.46, in Pearl River Delta of China, respectively^42^. However, it was much higher than the report by Huang, who observed the Cd-THQ and Pb-THQ of 0.23 and 0.11 (n = 248) in Zhejiang province, respectively^43^. The difference between the THQ values could be due to the effective parameters on THQ and their variations in different geographic locations.

**Table 6:** Estimated daily intake by human beings and potential health risk of heavy metals due to rice consumption.

	RfD^a^	M-EDI^b^				E-RfD^c^			
		Dongyang	Jiangshan	Longyou	Mean	Dongyang	Jiangshan	Longyou	Mean
Cd	1	0.56	1.22	1.06	0.92	9.7%	45.0%	29.4%	25.6%
Pb	3.5	1.36	0.84	1.71	1.38	3.2%	0%	5.9%	3.5%
Ni	40	2.96	2.86	6.84	4.5	0%	0%	3.0%	1.2%
Cr	1,500	1.23	0.91	2.07	1.5	0%	0%	0%	0%
Zn	300	101.5	105.8	136	116.5	0%	0%	0%	0%
Cu	50	15.7	14.5	18.2	16.4	0%	0%	0%	0%

	RfD^a^	THQ^d^				HI^e^			
		Dongyang	Jiangshan	Longyou	Mean	Dongyang	Jiangshan	Longyou	Mean
Cd	1	0.563	1.218	1.064	0.918	1.680	2.172	2.543	2.141
Pb	3.5	0.390	0.240	0.488	0.394				
Ni	40	0.074	0.072	0.171	0.112				
Cr	1,500	0.001	0.001	0.001	0.001				
Zn	300	0.338	0.353	0.455	0.388				
Cu	50	0.313	0.290	0.363	0.328				

^a^Reference oral dose (μg kg^−1^ day^−1^) according to EPA’s Integrated Risk Information System.

^b^Arithmetical mean estimated daily intake (μg kg^−1^ day^−1^).

^c^The percentage of heavy metals in rice grain samples which exceed the RfD.

^d^Ratio between EDI and the RfD.

^e^Sum of THQs.

Furthermore, the difference of health risk of the three counties was also compared in this study. The Cd-THQ of Jiangshan and Longyou were all higher than 1 and about 2 times higher than that of Dongyang. This result indicated the inhabitants in Jiangshan and Longyou were suffered more adverse health effects by consuming the contaminated rice. This difference was mainly due to the diverse topography and distribution of mining industry of the three counties. Jiangshan and Longyou are mountainous counties, and Shangfang mine^29^ and Suichang mine^30^ are all closed to these two counties. In contrast, Dongyang has relatively less mining factory due to its plain topography, which lead to less contamination in this area.

This Cd-THQ analysis was concurred with the risk evaluation that 34% of the rice samples exceed Chinese standard limit on Cd level in rice grains (0.2 mg kg^−1^), which was much more stringent than those other heavy rice-consuming countries such as Japan and Taiwan (0.4 mg kg^−1^)^44,45^. In fact, this risk assessment was appropriate for China because it was measured not only based on Cd levels in rice but also based on rates of rice consumption. According to the report^3^, populations in China showed much higher rates of rice consumption (238 g day^−1^) than those in Japan and Taiwan (119 and 132 g day^−1^, respectively). Furthermore, the actual rate of rice consumption in the Jin-Qu Basin was expected to represent roughly 1.5 of the national average^3^. Therefore, even a standard of 0.2 mg kg^−1^ might be inadequate for this region.

Pb, Zn and Cu contamination in rice also posed potential threats to local populations. Taken together, the value of hazard index (HI) for the consumption of rice in the Jin-Qu Basin was 2.141 (Table 6), indicating that local inhabitants may experience serious adverse health effects as a result. Relative contributions of Cd, Pb, Ni, Cr, Zn and Cu to the HI were recorded as 40.9%, 17.5%, 10%, 0.0%, 17.3%, and 18.2%, respectively. As micronutrients essential to human health, moderate levels of Cr, Ni, Zn and Cu found in the rice samples might not be a priority health concern^46,47^. However, it was necessary for local inhabitants to lower their levels of rice consumption and to diversify their diets to mitigate health risk associated with dietary Cd and Pb intake.

## Conclusions

The paddy soils in Jin-Qu Basin were characterized by acid and carbon rich properties and primarily contaminated by Cd. There were 34% and 30% of rice grain samples present higher levels of Cd and Pb in this study than the standard values of 0.2 mg kg^−1^, respectively. The spatial distribution maps for Cd and Pb revealed the similar geographic trends. The risk estimation of the dietary intake of heavy metals suggested that rice consumption poses serious health threats to the local population. This report can be used as an important reference for guiding policy makers in focusing on agricultural management and structural adjustment in this region.

## Materials and methods

### The study area

**Figure 2:**
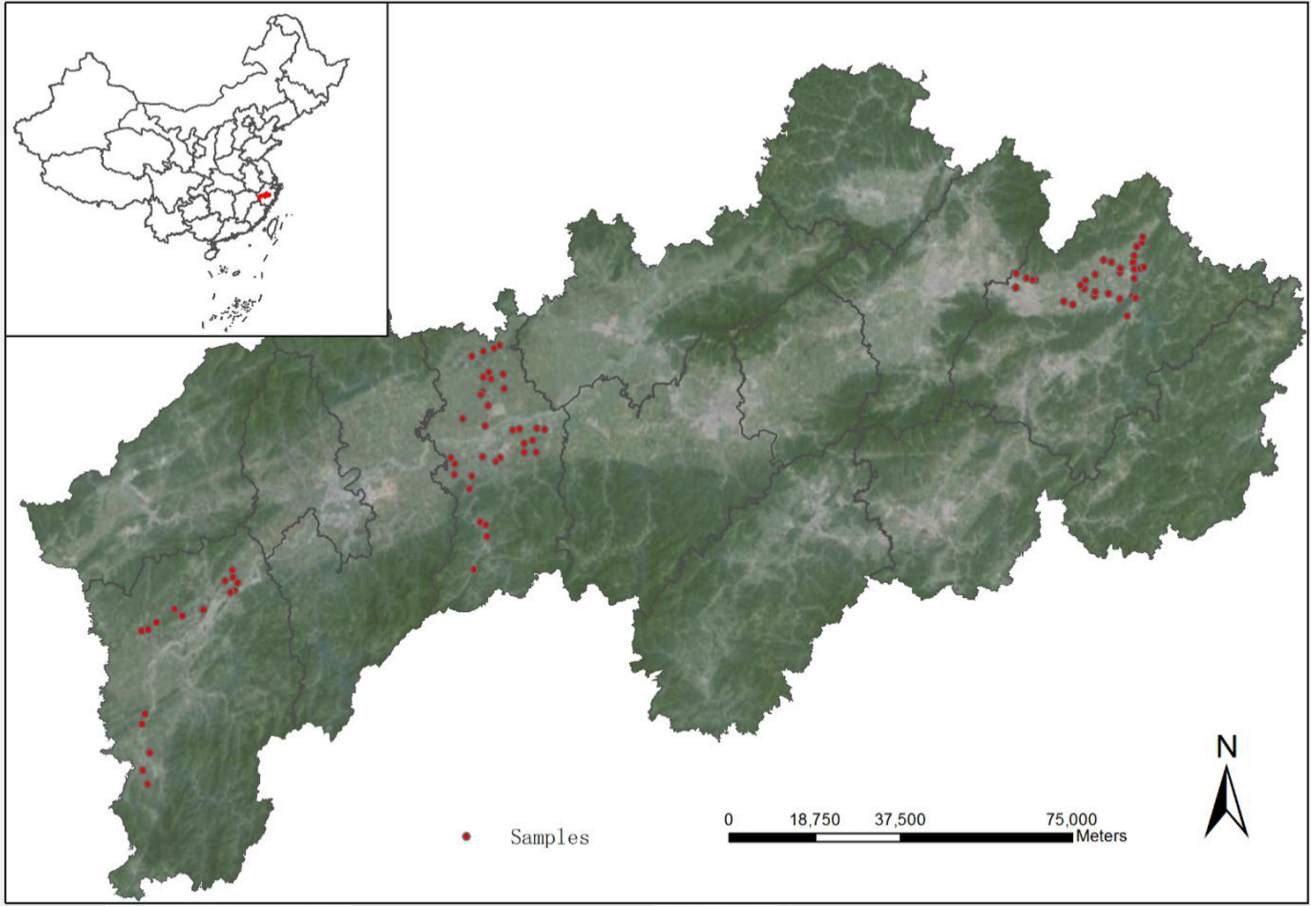
The location of study area and distribution of sampling points (The image was collected from Google Earth 7.3.2, https://earth.google.com).

Our survey was conducted in the Jin-Qu Basin, which is located in the centre of Zhejiang province (Fig. 2). It covers 118°01′ to 120°46′ E longitude and 28°15′ to 29°41′ N latitude and includes an agricultural area of approximately 3,700 km^2^ and a population of 8.14 million. The region is characterized by a subtropical climate with annual mean temperatures of 17.4 °C and rainfall levels of 1,500 mm. They are characterized by profound modification through human activities. Paddy soil belong to Acrisols and anthrosols due to aquatic rice production. The growing period for rice plants (*Oryza sativa* L.) runs from June to October. The soil is subjected to alternating flooded and dry conditions throughout the growing season. The main fertilizers are applied include urea, calcium superphosphate and potassium chloride.

### Sampling

Sampling sites were selected from arable land and plains of rice producing areas. Three typical areas of the Jin-Qu Basin are studied: the eastern (Dongyang county), central (Qujiang county), and western areas (Jiangshan county). These three counties have a long history of agricultural production, including approximately 11,000, 24,000, and 36,000 ha of developed and cultivated paddy land, respectively^9^. A total of 109 samples of topsoil (0–20 cm) and rice grain each were collected from the same locations under the harvest season (October, 2018). Each sample was collected as a composite of 5 sub-samples drawn over a distance of 5 m around each sampling site. The soil samples were thoroughly mixed, air dried at ambient temperature, passed through 2- and 0.149-mm mesh sieves, and then stored at ambient temperature prior to chemical analysis. For the rice samples, white rice grains were separated from rice hulls using a mill, ground into powder and stored at ambient temperature for heavy metals analysis.

### Analysis

Soil pH was measured at a solid:water ratio of 1:2.5 (w/v). SOM content was determined using the chromic acid titration method^17^. To analyse total concentrations of heavy metals, 0.1 g of ground soil samples was digested with 7 mL HNO_3_ + 2 mL HF + 1 mL H_2_O_2_ in a microwave oven (Mars 6, CEM Corporation, USA). Available heavy metals in the soil samples were extracted using the Bureau Communautaire de Référence (BCR) method^48^ whereby 1.0 g of air-dried soil (< 2 mm) was shaken for 24 h with 40 mL of a solution containing 0.11 mol L^−1^ acetic acid. Then, the liquid extract was separated from the solid residue through filtration, and the heavy metals in the extract were considered as available heavy metals. For the rice, 0.25 g of grain powder was digested with 5 mL HNO_3_ and deionized to a fixed volume.

Concentrations of Cd, Pb, Ni, Cr, Zn and Cu in the soil and rice grain samples were measured by inductively coupled plasma mass spectrometry (ICP-MS, Agilent 7,800, USA). Quality assurance and quality control (QA/QC) was verified from Chinese standardized reference materials: GBW07456-CSS-27 for the soil samples and GBW10023-CSB-14 for the rice grain samples. All samples were determined in duplicate. The blank and standardized reference materials were included with every 20 samples in the analyses. While applying these procedures, analytical quality control tests revealed high levels of precision throughout. The elemental recoveries and relative standard deviation (RSDs) for standardized reference materials were 96–105% and < 3.5%, respectively.

BAF was measured by the ratio between the heavy metals level in paddy soil and that measured in rice grains^49^.

### Human risk assessment

#### Estimated daily intake (EDI)

The estimated daily intake (EDI) of heavy metals via rice consumption was calculated using the following formula^50^:

$${\text{EDI}} = ({\text{FIR}} \times {{\text{C}}}) /{\text{ BW}},$$

FIR is the rice ingestion rate for an adult at the value of 238.3 g person^−1^ day^−1^. C is the concentration of metals found in the rice grain samples. BW is an individual’s body weight for an adult at 60.6 kg^4^.

#### Target hazard quotient (THQ)

The THQ is used to estimate potential health risks associated with the long-term consumption of contaminated rice. This method was developed by the Environmental Protection Agency (EPA). The THQ is the ratio between EDI and the reference oral dose (RfD) for each heavy metal as calculated with the following equation:

$${\text{THQ}} = {\text{EDI}}/{\text{RfD}},$$

The RfD values of the heavy metals, including for Cd, Pb, Ni, Cr, Zn and Cu, were recorded as 1.0, 3.5, 40, 1,500, 300, and 50 μg kg^−1^ day^−1^, respectively, according to the EPA^51^. When the THQ is valued at less than one, no obvious risks are posed. Conversely, when it is valued at equal to or greater than one, the exposed population will experience health risks^52^.

#### Hazard index (HI)

Exposure to more than two pollutants can result in additive effects^23^. Therefore, to measure potential risks of adverse health effects posed by the presence of heavy metals in rice, a HI approach was developed based on the EPA’s Guidelines for health risk assessment of chemical mixtures. This parameter, defined as the sum of THQs, is calculated as follows:

$${\text{HI }} = {\text{THQ}}_{{1}} + {\text{ THQ}}_{{2}} + {\text{THQ}}_{{3}} + \cdots + {\text{THQ}}_{{\text{n}}}$$

### Data analysis

All data were analysed with Microsoft Excel 2016 and SPSS 17.0. A Pearson correlation analysis and principal component analysis (PCA) were run to determine the relationships between heavy metals and the examined soil properties (pH and SOM). The level of significance was set to p < 0.05 or p < 0.01. The spatial distribution mapping of heavy metal concentrations in paddy soils was performed with ArcGIS 10.5 using ordinary Kriging.

## Supplementary information

Supplementary Information.
