# Supplementary material for: Health risk assessment of heavy metal pollution in a soil-rice system: a case study in the Jin-Qu Basin of China
Source: Sci Rep. 2020 Jul 13;10:11490. doi: 10.1038/s41598-020-68295-6 (PMC7359352; doi:10.1038/s41598-020-68295-6)
Supplement: Supplementary file 1 — Supplementary Information. [file 41598_2020_68295_MOESM1_ESM.pdf]

Health risk assessment of heavy metal pollution in a soil-rice system: A case study in the Jin-Qu Basin of China

Bin Guo[1 Δ *] Chunlai Hong[1 Δ] Wenbin Tong[3] Mingxing Xu[2] Chunlei Huang[2] Hanqin Yin[2] Yicheng Lin[1] Qinglin Fu[1*]

[1]Institute of Environment, Resource, Soil and Fertilizer, Zhejiang Academy of Agricultural Sciences, Hangzhou, 310021, China

[2]Technological Innovation Center for Arable Land Assessment and Restoration of Ministry of Natural Resources, Hangzhou, 311203, China

[3] Qujiang District Agricultural and Rural Burea, Quzhou, 324022, China

[Δ] Bin Guo and Chunlai Hong contributed equally to this article

[*] Corresponding author:

Bin Guo; Email: ndgb@163.com;

Qinglin Fu; Email: fuql161@aliyun.com

Figure legends

Supplementary Fig. S1 Histograms of soil Cd, Pb, Ni, Cr, Zn, Cu and soil pH, SOM

Supplementary Fig. S2 Bio-accumulation factor (BAF), a ratio of heavy metals concentration in rice grain to that in the corresponding soil in Jin-Qu basin

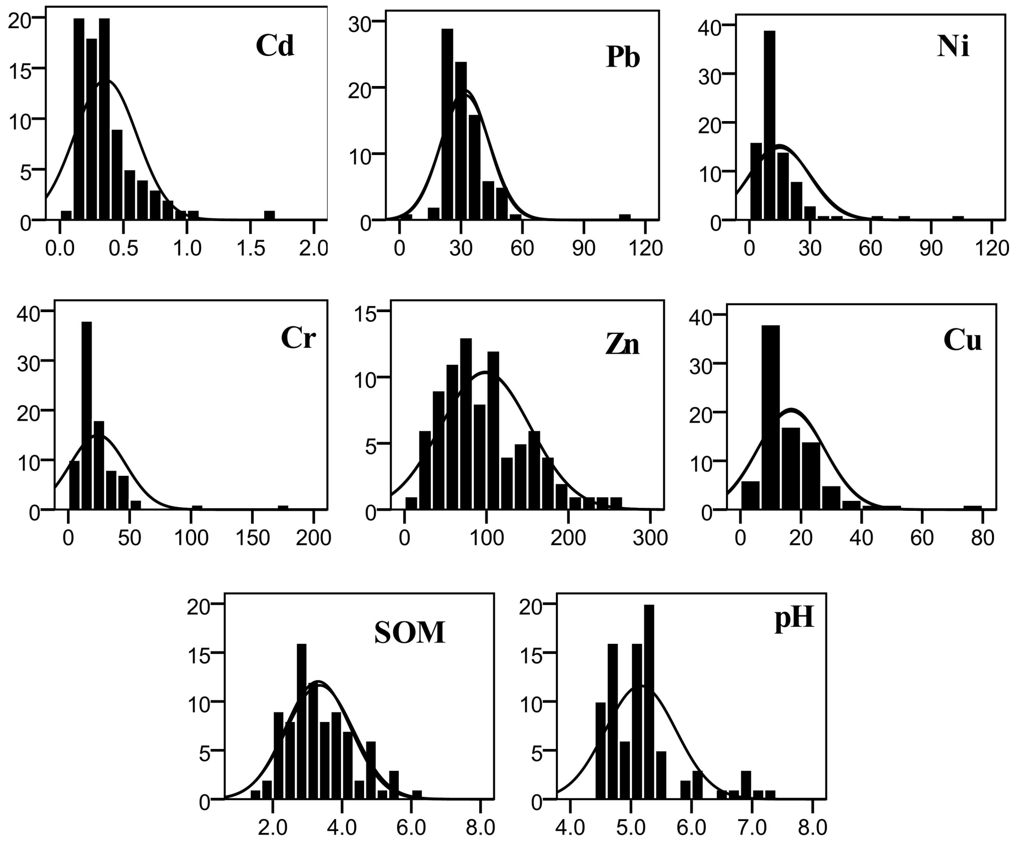

Supplementary Fig. S1 Histograms of soil Cd, Pb, Ni, Cr, Zn, Cu and soil pH, SOM

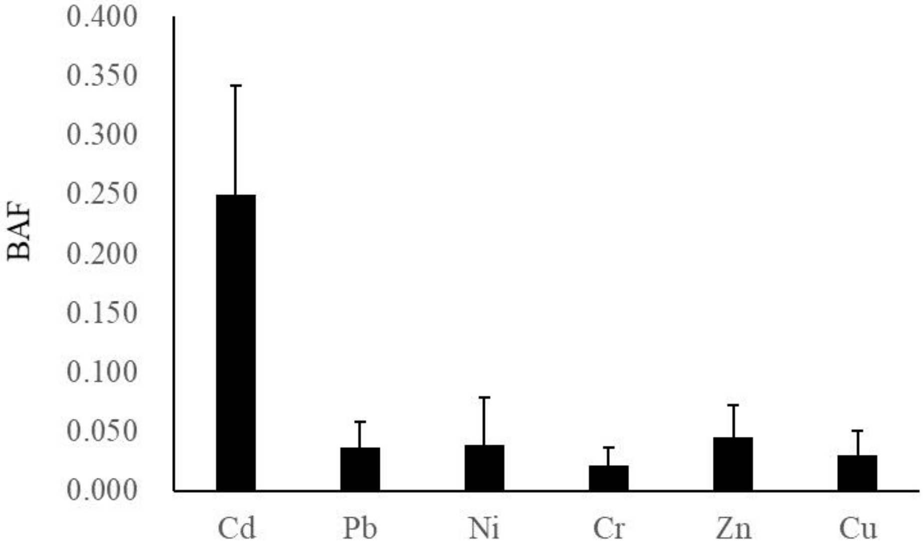

Supplementary Fig. S2 Bio-accumulation factor (BAF), a ratio of heavy metals concentration in rice grain to that in the corresponding soil in Jin-Qu basin
